# A Model for Training Public Health Workers in Health Policy: the Nebraska Health Policy Academy

**DOI:** 10.5888/pcd11.140108

**Published:** 2014-05-15

**Authors:** Kathleen Brandert, Claudine McCarthy, Brandon Grimm, Colleen Svoboda, David Palm, Jim P. Stimpson

**Affiliations:** Author Affiliations: Kathleen Brandert, Claudine McCarthy, Brandon Grimm, University of Nebraska Medical Center, College of Public Health, Omaha, Nebraska; Colleen Svoboda, David Palm, State of Nebraska, Department of Health and Human Services, Lincoln, Nebraska.

## Abstract

There is growing recognition that health goals are more likely to be achieved and sustained if programs are complemented by appropriate changes in the policies, systems, and environments that shape their communities. However, the knowledge, skills, and abilities needed to create and implement policy are among the major needs identified by practitioners at both the state and local levels. This article describes the structure and content of the Nebraska Health Policy Academy (the Academy), a 9-month program developed to meet the demand for this training. The Academy is a competency-based training program that aims to increase the capacity of Nebraska’s state and local public health staff and their community partners to use public health policy and law as a public health tool. Our initiative allows for participation across a large, sparsely populated state; is grounded in adult learning theory; introduces the key principles and practices of policy, systems, and environmental change; and is offered free of charge to the state’s public health workforce. Challenges and lessons learned when offering workforce development on public health policy efforts are discussed.

## Introduction

Momentum is building among public health workers around the notion that progress on public health goals is more likely to be achieved and sustained if programs are complemented by appropriate changes in the policies, systems, and environments that shape their communities (1–8). The Centers for Disease Control and Prevention actively encourages local and state grantees to consider policy-, systems- and environmental-change mechanisms, where appropriate, to address issues such as obesity and chronic disease (9,10). However, the knowledge, skills, and abilities needed to create and implement policy are among the major needs identified by practitioners at both the state and local levels (11,12).

We set out to develop an educational initiative, the Nebraska Health Policy Academy (the Academy), to increase the capacity of local and state public health personnel to use policy approaches in their work. The initiative had to allow for participation across a large, sparsely populated state (Nebraska); be grounded in adult learning theory (eg, clear purpose, self-directed learning, incorporation of real-life experiences); introduce the key principles and practices of policy change; and be offered free of charge to the state’s public health workforce (13). This article describes the structure and content of the Academy, as well as the challenges and lessons learned when offering workforce development on public health policy efforts.

## Conceptual Framework and Core Competency Development

The Academy is a competency-based training program that aims to increase the capacity of Nebraska’s state and local public health staff and their community partners to use public health policy and law as a public health tool. During the initial pilot year of the Academy, succinct domains and competencies, based on the Council on Linkages Framework (14), were developed in collaboration with health department staff and professionals with expertise in health policy and adult learning strategies and refined into a 6-stage Nebraska Health Policy Curriculum Framework. This framework (Table 1) was adapted from ideas and lessons learned from other health policy training programs and initiatives (14–17). Each of the framework’s 6 policy-making stages addresses a fundamental question encountered in the policy process. Several competencies are listed under each stage, and those competencies are the focus of the training. Although the framework appears linear, the policy process, in practice, may jump forward and backward through the stages.

**Table 1:** Competency-based Framework for the Nebraska Health Policy Academy

Stage 1	Stage 2	Stage 3	Stage 4	Stage 5	Stage 6
Who is involved and how?	What is the nature of the issue?	What will be done?	How to get the policy authorized?	How to put the policy into practice?	Did the policy make a difference?
Solicit community input when developing policies.	Define the problem requiring a solution.	Create a policy proposal.	Formulate a communication strategy.	Promote successful implementation of the policy.	Evaluate the impact of the policy change.
**Competencies**					
1A: Identify and assess the strengths and motivations of key stakeholders and potential resistors.	2A: Collect and summarize data on the public health burden, contributing factors, and health equity issues.	3A: Critique the feasibility and expected outcomes of potential policy options.	4A: Engage decision-makers.	5A: Assist with developing rules, guidelines, and procedures.	6A: Monitor outcomes of policy changes.
1B: Recognize and effectively use common styles for influencing others.	2B: Calculate the societal costs.	3B: Recommend a specific policy change.	4B: Frame messages and adapt materials to specific audiences.	5B: Educate the public about the policy change.	6B: Document whether the policy solution is functioning as intended.
1C: Build consensus on key values and a shared vision for action.	2C: Survey the social, economic, and political landscape.	3C: Calculate costs/returns of the policy change.	4C: Deploy coalition members in advocacy roles.	—	6C: Incorporate evaluation findings into future policy efforts.
—	—	—	4D: Advocate for resources needed to implement the policy.		—

## Implementation of the Academy

The Academy is focused on capacity development among teams of people with common interests. We believed the team model of learning would be more effective than an individual model for these types of participants and the goals of the training and also be more likely to have measurable outcomes at an organizational or community level (18–21).

Following a recruitment and application process, teams are accepted to participate in the training program. Teams of 5 to 7 members are composed primarily of state or local health department staff members and their key community partners; in particular, we encourage teams to include an elected official when possible. During the course of 9 months, these teams participate in a series of learning activities focused on the Academy competencies. Activities are a mixture of synchronous and asynchronous learning methods, as well as in-person and distance-based learning. There are 3 on-site sessions, live webinars and conference calls with guest faculty from across the country, and resources for self-paced learning via online modules and discussion boards. The topics have ranged from how to conduct cost-benefit analyses to strategies for marketing a policy solution to the general public and elected officials (Table 2). The variety of learning methods allows for exposure and access to those leading the policy field in Nebraska and nationally.

**Table 2:** Nebraska Health Policy Academy Example Curriculum by Stage

Stage	Mode	Example Curriculum
**Stage 1:** Who is involved and how?	Personal Assessment	Influence Style Indicator. Understand preferences and related tactics for influencing others (http://www.influencestyleindicator.com).
	Presentation	Coalition Building: Building Consensus and a Shared Vision for Action
	Assignment	Identify the potential proponents and opponents of the issue.
	Presentation	Getting Evidence into Policy
**Stage 2:** What is the nature of the issue?	Presentation	Finding Data on Your Issue: The County Rankings and Roadmaps Project
	Case study and discussion forum	“Coffee and Cigarettes.” Discuss the context of the problem and identify the level of policy change needed.
	Assignment	Write a data brief detailing the issue to be addressed.
	Asynchronous Web module	Cost Analysis Basics
**Stage 3:** What will be done?	Presentation	Understanding the Difference Between Programs and Policies
	Exercise	Stakeholder Power Analysis. Identify feasibility of policy options.
	Presentation	Policy in Action: Policy Changes Around Sugar-Sweetened Beverages
	Assignment	Write a policy proposal making the case for a recommended policy change.
**Stage 4:** How to get the policy authorized?	Presentation	Using the Four Political Values to Influence the Policy Process
	Assignment	Create a communication piece for your recommended policy using the skills of message framing.
**Stage 5:** How to put the policy into practice?	Presentation	Implementing Enacted Policies
	Asynchronous Web module	The Pieces of Policy Implementation
**Stage 6:** Did the policy make a difference?	Presentation	Evaluating Policy Interventions
	Asynchronous Web module	Identifying Measures for Evaluating Policy

As a core component of the Academy, using principles of adult learning, teams apply the knowledge and skills they receive to a policy development project that anchors the learning (22). The policy can be either a hypothetical issue in a community or a real-life public health problem that must be addressed. Additionally, teams can choose the level at which the policy change occurs: either at the governmental/legislative level or the organizational level. Examples of policy development projects from the Academy include developing a bill to implement statewide rules and regulations for community health workers, adopting alcohol and drug policies in public high schools, and seeking a local ban on sugar-sweetened beverages sold in vending machines on public property. Teams work on their project throughout the training period and receive one-on-one technical assistance on their policy development project from Academy staff and policy experts.

To date, 6 teams have completed the Nebraska Health Policy Academy (3 in each of the first 2 cohorts). There are currently 3 teams participating in the third cohort of the Academy. Teams’ members have ranged in number from 4 to 8 across the 3 cohorts. Members have included health department staff (directors, mid-level management, project coordinators), local elected officials (city council), partners from business and education (chamber of commerce representative, high school principal, community college representative), nonprofit partners, and clinicians, among others. Team composition depends on the needs of the policy development project undertaken.

The Academy shares similarities and differences with other training efforts on policy development. One well-known national effort, Shaping Policy for Health, a competency-based training program offered through the Directors of Health Promotion and Education, highlights how the Academy offers a unique opportunity (23). While both training programs offer curriculum geared toward assisting the public health workforce in understanding policy development, numerous distinctions can be made, 3 of which are significant. First, the structure of the 2 training programs differs. The Academy is offered as a continuous learning opportunity over 9 months. Each stage (Table 1) is addressed comprehensively during the 9 months, with multiple stages in play at one time. The Academy’s curriculum is a combination of face-to-face and distance learning. The Shaping Policy for Health curriculum is a series of face-to-face workshops, most of which are 2 days in length; each workshop covers only one large topic area.

Second, the Academy is designed to build strong partnerships within a given community by offering a team-based experience. You cannot come through the Academy alone but must be part of a group to address policy change. While Shaping Policy for Health is intended for audiences from one area at a host site, it is not required that the audiences form or serve as one team for the training. The third significant difference is in the function and participation of those teams. The Academy includes a hands-on, real-life experience for teams in that they must work on one policy development project relevant to their communities across the entire Academy experience. This gives teams a common problem to address together and tangible products to take with them when they leave the Academy, which they can then choose to use for community improvement. Shaping Policy for Health uses real-life case examples and activities but doesn’t require participants to take on a policy development project of importance to their communities as a part of the series of workshops.

Initial evaluation of the Health Policy Academy has included a pre- and post-test assessing change in knowledge of each of the 18 competency areas. Preliminary findings from cohorts 1 and 2 show an average increase in knowledge across all competencies of 47.9%, with a range of 29.6% (competency 6C) to 71.7% (competency 3A). Additionally, a focus group was conducted after the first year of the Academy, offering insight into the structure and function of the program. Creation of a comprehensive, empirical evaluation is underway to verify and add to the preliminary findings.

## Challenges and Lessons Learned

During the process of creating and running the Academy, we encountered challenges that may be unique to training public health professionals in policy development tools and techniques.

**Policy versus programs.** Our participants were unfamiliar with the difference between programs and policies. We now start with an interactive presentation that gives definitions and examples of how policies and programs differ yet together can solve community problems. Case studies are used to provide further training and examples on successful public health policies.

**Political sensitivity.** We found it challenging to balance the need for adult education that requires real-world application with the need to be sensitive to the political environment of each team. Adding to the complexity of the issue, public health organizations receive funding that often limits advocacy activities, and determining which boundaries are related to advocacy versus policy development can be difficult. We make it clear that the project developed in the Academy does not have to be implemented and should instead be viewed as a learning opportunity.

**Instructional methods. **We had to balance the participants’ needs for in-person time and technical assistance, both of which are resource intensive but valuable modes of instruction, with the limited funding resources that are typical of project grants. We achieved this balance by providing regular distance and asynchronous opportunities to maintain the training and team relationships.

## Conclusion

The Nebraska Health Policy Academy has seen success stories in fostering policy development and implementation. For example, one team worked on a project to reduce access to sugar-sweetened beverages in public facilities in the 3-county health district they serve. The policy to provide healthy vending machines was successfully implemented in local schools and has been expanded to encompass other public facilities including county government buildings and college and university properties. Success stories like these highlight the value of the Academy to health departments wishing to implement evidence-based policy (24).

We strongly encourage additional public health entities, including health departments, community advocacy groups, and non-profits focused on population health, to seek opportunities, such as the Nebraska Health Policy Academy, to learn how to effectively use policy development as a public health tool. Furthermore, because of the nature of the topic at hand — policy development — Schools of Public Health or other nongovernmental entities in other states can serve as neutral conveners of this type of training.

Our next goal is to develop a comprehensive, empirical evaluation of the Academy based on the Kirkpatrick Model that we can use to adapt and transform it (25). The intent is to have the evaluation capture changes in knowledge and skills, as well as monitor any policy changes made or implemented as a result of team participation in the training. We hope that the outcome of the evaluation will provide empirical evidence that we are arming public health professionals with another tool to sustainably improve population health.

## References

[1] Woolf SH . A closer look at the economic argument for disease prevention. JAMA 2009;301(5):536–8. 10.1001/jama.2009.51 19190319

[2] Smedley BD , Syme SL . Promoting health: intervention strategies from social and behavioral research. Washington (DC): National Academies Press; 2000. 25057721

[3] Flores LM , Davis R , Culross P . Community health: a critical approach to addressing chronic diseases. Prev Chronic Dis 2007;4(4):A108. 17875252 PMC2099273

[4] Stunkard AJ . Obesity and the social environment: current status, future prospects. Ann N Y Acad Sci 1977;300:298–320. 10.1111/j.1749-6632.1977.tb19331.x 279276

[5] McGinnis JM , Williams-Russo P , Knickman JR . The case for more active policy attention to health promotion. Health Aff (Millwood) 2002;21(2):78–93. 10.1377/hlthaff.21.2.78 11900188

[6] Frieden TR . A framework for public health action: the health impact pyramid. Am J Public Health 2010;100(4):590–5. 10.2105/AJPH.2009.185652 20167880 PMC2836340

[7] Hahn EJ . Smokefree legislation: a review of health and economic outcomes research. Am J Prev Med 2010;39(6):S66–76. 10.1016/j.amepre.2010.08.013 21074680

[8] Glassbrenner D . Safety belt use in 2003: use rates in the states and territories. DOT HS 809 713 Technical Report. Washington (DC): National Center for Statistics and Analysis, Advanced Research and Analysis, National Highway Traffic Safety Administration, US Department of Transportation; 2004.

[9] Bunnell R , O’Neil D , Soler R , Payne R , Giles WH , Collins J , Fifty communities putting prevention to work: accelerating chronic disease prevention through policy, systems and environmental change. J Community Health 2012;37(5):1081–90. 22323099 10.1007/s10900-012-9542-3

[10] Nichols P , Ussery-Hall A , Griffin-Blake S , Easton A . The evolution of the STEPS program, 2003–2010: transforming the federal public health practice of chronic disease prevention. Prev Chronic Dis 2012;9:E50. 22300870 10.5888/pcd9.110220 PMC3340214

[11] Dilley JA , Reuer JR , Colman V , Norman RK . From making pamphlets to making policies: results from a collaborative training to increase knowledge, motivation, and self-efficacy for achieving public health policy and systems change. Health Promot Pract 2009;10(2 Suppl):138S–45S. 10.1177/1524839909332601 19454760

[12] Nayar P , Opoku S , Apenteng B , Nuguyen A . Public health training needs in Nebraska: a mixed methods stakeholder assessment. Omaha (NE): Nebraska Center for Rural Health Research; 2012.

[13] Knowles M . The adult learner: a neglected species. Houston (TX): Gulf Publishing Company; 1984.

[14] Council on Linkages between Academia and Public Health Practice. Core Competencies for Public Health Professionals; 2010. http://www.phf.org/resourcestools/Pages/Core_Public_Health_Competencies.aspx Accessed February 7, 2014.

[15] Policy, systems, environmental development framework. Greenwood Village (CO): Uncommon Solutions, Inc. http://www.uncommonsolutionsinc.com/ Accessed February 7, 2014.

[16] Liberal Arts Instructional Technology Service. Texas Politics, Bureaucracy, 3.3 The Policymaking Process. University of Texas at Austin, 3rd edition, Revision 88; 2009. http://texaspolitics.laits.utexas.edu/8_3_3.html Accessed February 7, 2014.

[17] Emery J , Crump C . Public health solutions through changes in policies, systems, and the built environment: specialized competencies for the public health workforce. Washington (DC): Directors of Health Promotion and Education; 2006.

[18] Day DV . Leadership development: a review in context. Leadersh Q 2000;11(4):581–613. 10.1016/S1048-9843(00)00061-8

[19] Kozlowski SWJ , Ilgen DR . Enhancing the effectiveness of work groups and teams. Psychol Sci 2006;7(3):77–124. 10.1111/j.1529-1006.2006.00030.x 26158912

[20] Van Velsor E , McCauley CD , Ruderman MN , editors. The Center for Creative Leadership Handbook of Leadership Development. 3rd edition. San Francisco (CA): Jossey-Bass Publishers; 2010.

[21] Grimm LB . A study measuring the effectiveness of public health leadership institutes: improving skills, instilling behaviors and the limitations that exist in moving towards outcome based results [dissertation]. Omaha (NE): University of Nebraska Medical Center; 2013.

[22] Marquardt MJ . Optimizing the power of action learning. Mountain View (CA): Davies-Black Publishers; 2011.

[23] Shaping Policy for Health. Washington (DC): Directors of Health Promotion and Education. http://dhpe.site-ym.com/?page=Programs_SPH. Accessed March 13, 2014.

[24] Brownson RC , Chriqui JF , Stamatakis KA . Understanding evidence-based public health policy. Am J Public Health 2009;99(9):1576–83. 10.2105/AJPH.2008.156224 19608941 PMC2724448

[25] Kirkpatrick DL , Kirkpatrick JD . Implementing the four levels: a practical guide for effective evaluation of training programs. San Francisco (CA): Berrett-Koehler Publishers, Inc; 2007.

